# Analysis of the Lifestyle of Spanish Undergraduate Nursing Students and Comparison with Students of Other Degrees

**DOI:** 10.3390/ijerph19095765

**Published:** 2022-05-09

**Authors:** María Angustias Sánchez-Ojeda, Concepción Roldán, Lucía Melguizo-Rodríguez, Elvira de Luna-Bertos

**Affiliations:** 1 Department of Nursing, Faculty of Health Sciences of Melilla, University of Granada, C/Santander 1, 52071 Melilla, Spain; maso@ugr.es; 2 Department of Statistics and Operations Research, Faculty of Health Sciences of Ceuta, University of Granada, C/Cortadura del Valle Sn C.P., 51001 Ceuta, Spain; iroldan@ugr.es; 3 Biomedical Group (BIO277), Department of Nursing, Faculty of Health Sciences, University of Granada, Avda. Ilustración 60, 18016 Granada, Spain; elviradlb@ugr.es; 4 Instituto Investigación Biosanitaria, ibs.Granada, C/Doctor Azpitarte 4, 4^a^ Planta, 18012 Granada, Spain

**Keywords:** lifestyle, university students, nursing students, health behaviour, health promotion, healthy habits

## Abstract

Background: Nursing students are exposed to concepts of healthy lifestyles while they are attending university. Objective: The aim of this study was to analyze whether nursing students have a healthier lifestyle than non-nursing students and to determine whether their behaviour is consistent with their beliefs. Methods: A cross-sectional study, with 293 university students was performed by using a validated questionnaire to measure beliefs and behaviour regarding health. Results: The lifestyle pattern of the nursing students evaluated was characterised by a high percentage of nurses with low levels of physical activity, poor balanced diet and smoking habits. The comparative analysis showed no significant differences between nursing students and students from other degrees. Conclusions: Students have a positive attitude and knowledge about healthy lifestyle, but do not transfer it to their own lives. Nurses’ lifestyle can unintentionally affect the behaviour of other people through their own behaviour and beliefs because they serve as a model for a healthy lifestyle. These findings support that nurse educators have an active role as promoter of health by using lessons to modify the behaviour of their students.

## 1. Introduction

Lifestyle refers mainly to everyday human behaviour that characterises an individual’s way of life and is usually permanent. Moreover, it includes the individual’s beliefs and knowledge about it. Healthy habits are not only healthy eating and daily exercise; there are also several other dimensions including substance abuse, sexuality, road safety, etc. [1,2,3]. A healthy lifestyle reduces the risk of being ill or early death [4].

In 2005, the National Council of State Boards of Nursing (U.S.A.) [5] recognised the importance of nursing competencies related to clinical practice in the context of public health. The teachers of the nursing degree have the responsibility to train the students in nursing competencies in this area during the degree. According to the Patient Protection and Affordable Care Act (U.S.A.) [6], it is necessary to emphasize the activities of prevention and health promotion in the community developed by nurses [7]. The implementation of educational strategies on public health during the theoretical and clinical training of students of the nursing degree can favour the adoption of healthy lifestyles among them, thus facilitating their subsequent application for the community approach [8].

Most university students fall in the age group of young adults, i.e., 18–24-year-old. This group is characterised as highly vulnerable because at this age they are affected by various factors, such as physiological, emotional, environmental changes, starting university, and living away from home for the first time, among other [9,10,11].

Recent surveys conducted confirm a rapid decline in healthy lifestyle habits within this group [9,11,12,13,14]. In particular, studies in the dietary patterns and lifestyle of the student population indicated that they are characterised by unhealthy diet, excessive alcohol consumption, smoking, and a sedentary lifestyle. These factors have a very significant impact on health, in the sense that their presence may constitute a risk factor for diseases such as coronary heart disease, diabetes, obesity, cancer, and osteoporosis, among others [15]. In particular, it has been observed that students from countries that have traditionally had a Mediterranean diet, a dietary pattern known for its health benefits, consume lower amounts of vegetables and fish while increase their intake of red meat and animal fats [12,16,17]. This corresponds to the globalized behaviour and belief amongst young people. Considering that at this age the patterns that remain in adulthood are acquired, different authors consider that promoting health and encouraging healthy habits in the young is a priority [18,19].

This problem particularly affects students of health sciences because once they finish their degree and become trained nurses, they will represent a model of healthy lifestyles for the promotion of health in the general population. In this regard, some studies have shown that patients and families give more attention to advice on healthy lifestyles than those who appear to follow it themselves [20,21,22,23]. They are responsible for transmitting, teaching, and modifying the behaviour of the general public towards a healthy lifestyle in order to prevent diseases in later life. Moreover, it is important that students who have taken these degrees must be prepared not only to apply their knowledge in their careers but also apply them to their daily lives for their own benefit and responsibility, the latter concerning their role as nurses in the promotion of a healthy lifestyle, a need that has increased in recent years as a consequence of increasing pathologies associated with a poor lifestyle [24,25,26,27]. However, despite the evidence that nurses who have a healthy lifestyle are able to be more motivating or generate more confidence in the patient [28,29], the reality is that our nurses, already from the university stage, do not apply the advice of healthy habits in their own practice [20,24,30].

In this context, it is important to assess the behaviour and beliefs of nursing students and to compare them with those observed in other university degrees. Thus, the aim of this study is to explore the lifestyle of nursing students and determine if the skills acquired in this degree are reflected in their own lives, if this lifestyle is healthier than the lifestyle of students from different degrees at the same campus, and to ascertain whether their behaviour is associated with their beliefs.

## 2. Materials and Methods

### 2.1. Sample

The present study was cross-sectional and conducted in the University of Granada, at the Melilla campus, located in the north of Africa. The population included all the students of the School of Nursing and a sample of students of humanities, education, and social sciences. A total of 293 students were evaluated.

### 2.2. Questionnaire and Protocol

A self-made questionnaire was used, which was previously validated among a group of people who fulfilled the same characteristics as our study population (see Supplementary Materials). The questionnaire was developed to collect data concerning health behaviour and beliefs on health-related behaviour. This study used the Delphi method to validate the final design of the questionnaire. A two-round Delphi iterative consultation process was performed with 11 experts. Our selection criterion was that they had to be university lecturers/professors or nursing professionals with a recognized academic career in the area of this research. After the first round, the expression of several items was modified due to the difficulty of understanding them. The questionnaire was validated after the second round. All questions were closed-ended with possible responses ranging from either a ‘Yes/No/Undecided’ (43 questions) or given by multiple choice options consisting of a set of ranges or values (21 questions). The first part of the survey comprised 38 questions in five sections: exercise; nutritional behaviour; consumption of tobacco, alcohol, and other drugs; sexual behaviour; and road safety. The second part consisted of 26 questions on exercise beliefs; nutritional beliefs; smoking, alcohol, and other drug use; sexual beliefs; and road safety.

The questionnaires were administered during the academic year, in November and December 2021, outside the examination period in order that participants’ stress state did not influence their answers. They were informed of the purpose of the study, and asked for their collaboration on an anonymous and voluntary basis with emphasis on the sincerity of their answers. The survey was distributed and completed during class time, either before or at the end of the class (allowing a minimum of 10 min for students to complete it), and was returned to the research coordinator.

### 2.3. Statistical Analysis

Data were coded and entered into the SPSS software (v.20 for Windows; IBM, Somers, NY, USA). Descriptive statistics (i.e., frequencies, percentages, central tendency measures, etc.) were used to describe the characteristics or the students on demographics and healthy lifestyle behaviour and beliefs. The level of statistical significance was set at *p* < 0.05 for all statistical tests. Comparisons between groups were performed using chi-squared tests with Yates’ correction. Odds ratio (OR) was used as a measure of association to quantify the relationship between the variables analyzed in nursing and non-nursing students, and between real lifestyle and health beliefs of the students. Principal components analysis (PCA) with varimax rotation was employed to show the underlying framework of data (seeking for interesting relationships between the variables).

### 2.4. Ethical Considerations

This study was approved by the Ethical Committee for Biomedical Research of Granada, Spain, (2578/CEIH/2022) and developed within the legal framework governed by the provisions of the Law on Protection of Personal Data 15/1999 and the General Health Act 14/1986 of Spain, on access to medical records for legal, epidemiological, public health, research, or teaching purposes. In accordance with this law, questionnaires were anonymous and only the sex and the age data were retained and a brief explanation of confidentiality and informed consent was given. The data collection was performed in accordance with the 1964 Helsinki Declaration (Ethical Principles for Medical Research Involving Human Subjects) and its seven later amendments.

## 3. Results

### 3.1. Description of Participants

The characteristics of the students are reported in Table 1 and Table 2. A total of 293 students completed the questionnaire; 166 were nursing students and 127 were non-nursing students belonging to humanities, education, and social science disciplines. The level of participation in the survey by students from the different faculties was approximately 20%. The number of students in their first-year was 153 (52.2%), 33 (11.3%) in their second-year, 73 (24.9%) in their third-year, and 34 (11.6%) in their fourth-year. Students were predominantly 18–25 years of age (88%) with only a few students older than 30 (3.1%). There was a higher ratio of females (67%) to males (33%), which is normal in the university degrees considered. Most students were Catholic (78.2%) and the others (21.8%) were Muslim.

Mean BMI was 23.94 (SD 3.88) and 22.49 (SD 3.31) in men and women, respectively. No significant gender differences were found on BMI, cigarette consumption/day, age at first alcohol use, and age at onset of sexual activity (*p*-value > 0.5 in all cases). In relation to exercise, 55.3% walk to university, 44% go by car, and only 0.7% use public transport. Most exercise, play sports, or walk (67.6%), but only slightly more than half (56.7%) say they have time for exercise. The largest group of students (35.2%) exercise 1–2 days a week, 17.4% 3 days, 8.5% 4 days, and 11.3% 5 days or more. Most (36.5%) do between 0.5 and 1 h of exercise per day (18.4% less than half an hour per day, 15% between 1 and 2 h, and only 3.1% more than 2 h). Of all respondents, 80.3% have stable weight and 61.3% drink 4 to 8 glasses of water a day. Approximately 60% eat sweets, less than 3 servings of milk, and less than 2 fruits per day, while 38.1% eat 2 or more servings of meat per day and 27.6% admitted to having followed some weight loss programme during the past year.

Results on tobacco consumption showed that 38.4% of students smoked cigarettes at the one time while only 18.9% currently smoke (10 is the most common number of cigarettes smoked per day, the average is 7.92, SD 4.58). Most (67.7%) allow smoking in their presence, and have drunk alcohol (69.6%). The average age of first alcohol consumption is 15.56 (SD 2.22). Considering the students who drink alcoholic beverages, 38.3% said that they drink every month and only 13.7% drink every day. The percentage of students who smoke marijuana or hashish is 31.4% (more than 3 per week is 16.2%); 1 student consumes solvents or LSD daily; 2 students take heroin or pills every day; and 3.4% use cocaine.

Half the students (55.6%) claimed to have a stable partner and 74.3% reported using contraception. The average age of onset sexual activity is 16.36 (SD 1.93). In relation to contraceptive methods, 30.3% of the participants use the day-after pill (DAP) and 43.6% have had unprotected sex at the one time.

Concerning road safety, 32.2% of the students have driven under the influence of alcohol or drugs, and 59.4% have travelled with someone driving under the influence of alcohol or drugs. Additionally, 40% of respondents reported having driven exceeding the speed limit and 53.8% use a mobile phone while driving.

Almost all students were aware of sexually transmitted diseases (STDs); wear a helmet when they go on a motorcycle or the seatbelt when travelling by car; believe that physical activity helps improve health, prevent disease and gives vitality but is boring; that drinking water is important for health; good nutrition improves academic performance; smoking is addictive; condoms prevent the acquired immune deficiency syndrome (AIDS); and unwanted pregnancy and accidents can be avoided.

In relation to beliefs, 13.7% of the students consider that it is healthier to be thinner while 15.5% think that their body is the most important. On the other hand, 60.4% believe that social drinking is normal and 40.1% find it enjoyable. In relation to beliefs about drugs, 83.5% of the subjects consider they have easy access to drugs and 74.5% believe they have sufficient information about drugs. For contraceptive methods, 71.4% think that condoms are the best method of contraception, but 49.6% believe that is uncomfortable. While 14% believe the DAP is similar to any other contraceptive method, about 33.3% consider it is better to enjoy sex without contraception. Finally, 55.1% of respondents consider accidents as a problem of health and life.

### 3.2. Comparison of Healthy Lifestyles and Beliefs between Undergraduate Nursing and Non-Nursing Students

The results of these comparisons are detailed in Table 3. Concerning exercise, the probability of having time to exercise is greater for students from other faculties than for nursing students (OR = 1.678, *p*-value = 0.042). Regarding nutritional behaviour, the probability of drinking 4 to 8 glasses of water a day is higher among nursing students (OR = 1.91, *p*-value = 0.011) and that of going to university without breakfast is greater for students from other faculties than for nursing students (OR = 2.083, *p*-value = 0.004). Concerning the consumption of tobacco, alcohol, and other drugs, the probability of ever having drunk alcoholic beverages is higher for nursing students (OR = 2.130, *p*-value = 0.000). The other comparisons were not significant. In particular, with respect to sexual behaviour and road safety, there is no difference between nursing students and those of other faculties.

The results of comparisons of beliefs about healthy lifestyles of undergraduate nursing students and non-nursing students are also presented in Table 3. Regarding exercise or nutritional behaviour, there is no difference. Concerning consumption of tobacco, alcohol, and other drugs, the probability of believing that drinking alcohol is normal in social situations is higher among nursing students (OR = 1.800, *p*-value = 0.021) and the probability to believe that drinking alcohol is fun is higher for the same group (OR = 2.150, *p*-value = 0.005). On the subject of road safety, the probability of believing that accidents are a health and life problem is higher among nursing students (OR = 2.287, *p*-value = 0.001) and the probability of believing that drugs affect driving is higher in this group of students (OR = 3.937, *p*-value = 0.002). The other comparisons were not significant.

### 3.3. Comparison of Healthy Lifestyles and Health Beliefs

The previous results show that only in a few cases the behaviour and beliefs of nursing and non-nursing students were significantly different. Therefore, the comparisons between health lifestyles and beliefs were performed on the full sample. Results are given in Table 4. For example, regarding exercise, the probability of doing any physical activity is higher for students who do not find exercise boring (OR = 3.23, *p*-value = 0.005). Note that regarding sexual behaviour, there is no difference between behaviour and beliefs.

PCA with a KMO (Kaiser–Meyer–Olkin) measure of 0.700 shows the need to use sixteen components for describing the data (these components explain 66.48% of the variability). The first nine factors are shown in Table 5. The first factor is related to smoking and shows that the consumption of hashish/marijuana is associated with smoking cigarettes. The second factor is related to drinking alcohol and shows that religion is associated with smoking and drinking. The third factor identifies those variables related to playing sports. The fourth factor represents drug consumption. The fifth shows that sex is associated with weight and height but also with the variable that asks whether sex is more enjoyable without any contraception. The sixth factor is related to healthy beliefs and habits. The seventh represents bad driving habits. The eighth factor considers unconscious behaviour related to youth and the ninth reveals the body image. The remaining factors are not of interest.

## 4. Discussion

A healthy lifestyle of the population has been shown to be the best way to avoid a future increase in cardiovascular disease or cancer [1,15]. In this study, different dimensions of lifestyles within the university population and the differences between students of a nursing degree and other degrees are discussed.

It is well known that exercise benefits physical and mental health as well as being a factor in the prevention of various diseases [31,32]. The literature shows that although university students have a high percentage of favourable beliefs related to physical activity and sport, this is not reflected in their actual physical activity, which is lower or not healthy [16,19]. The results obtained in this study regarding beliefs reflect that there is a large percentage of students who believe that exercise helps improve physical health and prevents disease, and that one feels better and with more vitality. However, the analysis of the behaviour shows a high percentage (72.4%) of students who do sports also exercised once or twice a week (35.2%) or exercised regularly (four or more days a week) (20%). These data differ greatly from the results obtained in a survey on university students in Madrid, Spain ([33]), where 40% exercised regularly (3–4 h/week average). Similarly, the results published in Moreno-Gómez et al. (2012) [15] showed that 85.7% of students exercised three or more hours per week.

It should be noted that the results obtained in relation to dietary habits among university students surveyed show an increase in the intake of meat products and a decrease in the number of recommended servings of fruit and vegetables. These data are consistent with that reported in other studies, which show a change in the diet towards unhealthy habits, with increased intake of meat products, snacks, sweets, and pastries, and a low consumption of fruit and vegetables [11,12,34,35]. This change may be due, as pointed out by various authors, to different factors (economic, social, and cultural), food preferences, and to the fact that they leave home for the first time to go to the university, among others [12,33,35]. In our study, it should also be noted that a significant proportion of the population analysed is Muslim, which may also represent a factor to be considered in the dietary patterns of these students. In contrast, some studies have shown favourable results concerning university students’ adherence to healthier dietary patterns such as the Mediterranean diet. In this regard, a study conducted in Navarra (Spain) between 1999 and 2010 showed a positive trend towards the daily consumption of fruit and vegetables [36]. Similarly, Rodríguez-Muñoz et al. reported high adherence to the Mediterranean diet among students at the Universities of Córdoba and Castilla-León (Spain) [37].

A clear trend is emerging in the student population. Currently, we find a large percentage of people who are unhappy with their body image and this leads them to dieting. Montero-Bravo et al. (2006) [33] observed that energy intake in the university population was below that recommended, and thought that this could be attributed to the fact that at this age people usually worry about maintaining or achieving a lean body. Furthermore, it should be noted that aspects related to social and emotional behaviours, especially in women, have worsened after the COVID-19 pandemic, as demonstrated by Ruiz-Zaldibar et al. in a study of university nursing students in Spain [38]. According to the results obtained in our study, those students who feel that the most important aspect is to be thin and have a good figure are those who eat 2–3 servings of meat daily. Moreover, these students are those who had dieted in the previous 12 months.

The percentage of students who smoke or have ever smoked (18.9%) is similar to those reported in other studies [33,36,39]. Rodríguez-Gázquez et al. 2017 [29], in a study carried out at the University of Seville (Spain) and the University of Antioquia (Colombia), observed that only 10% of the students included in the study were current smokers, which is a lower percentage than observed in our results. In our study, PCA showed that the consumption of hashish/marijuana was associated with smoking in the sense that it can be considered a soft drug. However, these figures are not only limited to nursing students, but a trend that extends to other health-related careers. In this sense, La Torre et al. observed that the overall prevalence of smoking among medical students was 29.3% (95% confidence interval 28.1–34.7), with percentages ranging from 28% in Germany to 31.3% in Italy [40].

In relation to alcohol consumption, our findings show that a high percentage of the students are frequent consumers of alcohol, results that are in line with those found by Moreno-Gómez et al. (2012) [15] who observed that 80% of the Spanish university students included in their study consumed alcohol either sporadically or habitually. They and others postulate that alcohol consumption is a behaviour ingrained in our society without being affected by other aspects of lifestyle. The data obtained confirm it since over 60% of students believe that drinking is normal in social situations. In the same vein, Aiello et al. compared the levels of alcohol consumption among university students in Spain and Italy, showing a higher alcohol consumption among Spanish university students [41]. However, it should be borne in mind that alcohol consumption in Spain is consistent with the model for countries in transition, where binge drinking is the most common pattern of excessive use of alcohol [42].

Regarding sexuality, the group studied has a good knowledge of STDs and recognises that the use of condoms is the best way to prevent STDs and unwanted pregnancy. The age of onset of sexual activity reported is similar to results in other published studies [9,34]. The main negative consequences for the health of university students are the spread of STDs and unwanted pregnancy. These risks are associated with early-age sexual initiation and alcohol and/or drug use. Results obtained agree with other studies which show that, even with information about STD prevention, they continue to expose themselves to risk [43].

Road safety, the last dimension analysed, shows that a high percentage of students surveyed wear helmets and seatbelts. It should be noted that the number of students who have ever driven under the influence of drugs or alcohol and who have travelled with someone in these conditions is statistically significant, because although most accidents occur during the day and on workdays, the proportion of young people who died during the night and weekends is much higher than for other ages.

Focusing on the comparative analysis between nursing and other students, in general, there are no significant differences. Our findings are in line with those obtained by Kritsotakis et al. who found no significant differences between the lifestyle habits of nursing students and students of other degrees from Greece [30]. These results could be due to factors such as the similarity in age of the participants or the unhealthy practices that begin to be acquired in these stages of life and are shared by individuals in this age range, regardless of the degree for which they are studying [44].

Nutritional habits were analysed by Rizo-Baeza et al. (2014) [24] in nutrition and nursing degrees, concluding that students of health sciences do not apply the knowledge that they acquire at university to their own nutritional habits. In our study, the nursing students do not have healthy habits. Even so, we should endeavour to change their behaviours since unhealthy habits such as poor diet sedentary lifestyle, smoking, alcohol consumption, etc., may interfere with their ability to serve as role models to the general public [39,45].

The promotion of healthy habits and the prevention of illness are considered within nursing competencies [8,46,47]. Currently, nursing interventions on lifestyle extend from face-to-face in medical appointments to telephone or telematic interactions [48,49]. Thus, nursing interventions developed from primary healthcare are able to positively influence the lifestyles of the population, favouring the prevention of chronic pathologies associated with unhealthy lifestyle habits [50]. Nursing students should become aware of the importance of following a healthy lifestyle, because in the future they will be, along with patients and other professionals, responsible for the acquisition of habits that will have a positive impact on the prevention and development of different pathologies. Despite the findings obtained in this work, several studies support the fact that the lifestyle of nursing students can be positively influenced through an adequate educational program [1,51,52]. According to these findings, other studies have shown that the lifestyle of nursing students vary throughout their nursing education, obtaining better results at the end of the degree [51]. University, therefore, is an appropriate environment to develop intervention strategies to improve the lifestyle of students and especially in degrees related to health sciences and nursing.

The main limitation of this study was its local character since it was developed on a campus from a small region of Spain. Despite this limitation, the population analysed is characterized by an important multiculturalism which strengthen this study. In any case, it would be interesting to expand the sample to other universities.

## 5. Conclusions

We conclude that the students have a positive attitude about healthy lifestyle but do not transfer it to their own lives. Therefore, it is not only necessary to have the knowledge, but it is also required and important to end unhealthy behaviour and lead the students towards a healthier lifestyle. Therefore, nursing degree teachers should address this problem to ensure that health professionals have the appropriate training to be able to transmit and educate the general public on a healthier lifestyle.

## Figures and Tables

**Table 1:** Sociodemographic characteristics of participants.

	Nursing (*n* = 166)	Humanities and Education (*n* = 102)	Social Sciences (*n* = 25)	All (*n* = 293)
Age (years)				
18–20	83 (50.0%)	55 (53.9%)	5 (20%)	143 (48.8%)
21–25	57 (34.3%)	41 (40.2%)	17 (68%)	115 (39.2%)
26–30	19 (11.4%)	4 (3.9%)	3 (12%)	26 (8.9%)
>30	7 (4.2%)	2 (2%)	0 (0%)	9 (3.1%)
Gender				
Females	117 (10.5%)	68 (66.7%)	10 (40%)	195 (66.7%)
Males	49 (29.5%)	34 (33.3%)	15 (60%)	98 (33.3%)

**Table 2:** Characteristics of participants by gender.

	Men (*n* = 98)		Women (*n* = 195)		All (*n* = 293)	
	Mean	SD	Mean	SD	Mean	SD
Weight (kg)	76.28	14.39	60.57	9.58	66.32	10.73
Height (cm)	177.05	7.40	163.33	168.35	168.35	9.39
BMI (kg/m^2^)	23.94	3.88	22.49	3.31	22.99	3.58
Cigarette consumption/day	7.48	3.98	8.23	4.99	7.92	4.58
Age at first alcohol use	15.78	2.28	15.55	2.20	15.56	2.22
Age at onset of sexual activity	16.59	1.51	16.17	2.13	16.39	1.93

**Table 3:** Lifestyle and health beliefs of participants.

Lifestyle Characteristics	All %	Nur. %	Non-Nur. %	*p*	Belief Characteristics	All %	Nur. %	Non-Nur. %	*p*
*Physical activity*					*Physical activity*				
I exercise or I walk	67.6	63.3	73.2	0.093	Weight-stable	99.9	100.0	97.6	0.160
I have time to do it	56.7	51.2	63.8	0.042 ^§^	It is boring	9.6	10.8	7.9	0.512
My weight is stable	80.5	78.9	82.7	0.511	It gives vitality	92.9	93.4	92.2	0.891
*Eating habits*					*Eating habits*				
4–8 glasses of water/day	61.4	68.1	52.8	0.011 ^§^	Thinner = Healthier	13.7	13.9	13.4	1.000
>2 sweet servings/week	59.4	59.0	59.8	0.985	Figure is the most imp.	15.4	13.9	17.3	0.514
<2 fruits/day	57.0	58.7	55.9	0.833	Water is imp. for health	98.6	99.4	97.6	0.436
<1 vegetable servings/day	39.6	35.5	44.9	0.134	It improves academic perf.	98.3	98.8	97.6	0.762
>2 meat servings/day	37.9	36.7	39.4	0.736	Fast food = More time	26.6	26.5	26.8	1.000
<3 milk servings/day	56.3	57.8	54.3	0.631	*Tobacco/Alcohol/Drugs*				
>2 cake servings/day	22.9	18.7	28.3	0.070	I would like to stop smoking	72.9	80.0	63.3	0.200
>2 salted snacks serv./day	56.7	52.4	62.4	0.119	I would ask for help to stop it	59.2	56.3	62.9	0.506
Diet in the last 12 months?	27.3	30.1	23.6	0.269	Smoking is addictive	95.7	98.0	92.5	0.065
I go to univ. without breakfast	38.2	30.7	48.0	0.004 ^§^	I smoke to not feel excluded	11.9	9.0	17.5	0.291
*Alcohol/Tobacco/Drugs*					Alc. is normal in social relations	61.4	67.5	53.5	0.021 ^§^
Have you ever smoked?	38.2	39.8	36.2	0.620	Alcohol is fun	40.1	48.1	30.1	0.005 ^§^
I currently smoke	18.8	18.7	18.9	1.000	I have easy access to drugs	83.5	83.8	83.2	1.000
I allow smoking	67.6	71.1	63.0	0.180	I have enough information	75.8	75.9	75.6	1.000
Hashish/marijuana?	31.4	34.5	27.2	0.227	Drugs are dangerous	97.5	98.2	96.7	0.719
I have drunk alcohol	69.6	78.9	57.5	0.000 ^§^	*Sex*				
I take cocaine/heroin daily	3.4	3.6	3.2	1.000	*Condoms:*				
*Sex*					-are the best choice	73.4	77.1	68.5	0.129
I have a stable partner	55.6	58.2	52.1	0.371	-are uncomfortable	49.6	48.3	51.5	0.713
I use contraception	74.3	76.9	70.2	0.351	-prevent AIDS	93.9	94.6	92.9	0.732
I know about STDs	98.3	99.4	96.6	0.225	-prevent unwanted pregnancy	95.2	97.0	92.9	0.179
I use DAP as contraception	30.3	26.2	36.4	0.144	DAP is like any other method	14.0	10.7	18.8	0.088
I have had unprotected sex	43.6	46.4	39.6	0.356	Sex is better without contraception methods	29.0	25.3	33.9	0.142
*Road* *safety*					I have good sexual information	92.4	93.2	91.3	0.730
I wear a seatbelt/helmet	97.3	97.6	96.9	0.981	*Road* *safety*				
I have driven drunk/drugged	31.4	33.7	28.3	0.391	*Accidents*				
I have travelled with someone under the influence of alcohol/drugs	59.4	65.5	51.2	0.021 ^§^	-are preventable	97.9	97.6	98.4	0.933
I drive exceeding the speed limits	39.2	41.8	36.2	0.419	-are a health and live problem	56.3	65.1	44.9	0.001 ^§^
I drive using a mobile	54.6	55.4	53.5	0.840	Drugs affect driving	86.9	81.2	94.4	0.002 ^§^

^§^: *p* < 0.05.

**Table 4:** Odds ratio for lifestyle and health beliefs of the students.

Lifestyle	Believes	OR	95% CI (OR)
*Physical activity practice*			
I exercise or I walk	It is not boring	3.23	1.46, 7.14
I exercise or I walk	It gives vitality	4.10	1.58, 10.66
I have time to do	It gives vitality	4.40	1.54, 12.62
*Eating habits*			
>2 meat servings/day	Thinner = Healthier	2.21	1.13, 4.34
>2 meat servings/day	Figure is the most important	2.48	1.29, 4.75
<3 milk servings/day	Figure is the most important	2.29	1.18, 4.43
Diet in the last 12 months?	Thinner = Healthier	2.74	1.37, 5.52
Diet in the last 12 months?	Figure is the most important	2.28	1.18, 4.43
I go to univ. without breakfast	Thinner = Healthier	2.20	1.12, 4.32
*Tobacco/Alcohol/Drugs*			
Have you ever smoked?	Drinking alcohol is normal in social situations	3.08	1.80, 5.26
Have you ever smoked?	Drinking alcohol is fun	4.63	2.73, 7.85
I currently smoke	I would ask for help to stop it	6.14	2.98, 12.66
I currently smoke	Drinking alcohol is normal in social situations	2.40	1.21, 4.75
I currently smoke	Drinking alcohol is fun	3.78	2.00, 7.14
I smoke hashish/marijuana	I would ask for help to stop it	2.09	1.09, 4.00
I smoke hashish/marijuana	Smoking is addictive		
I smoke hashish/marijuana	Drinking alcohol is normal in social situations	2.84	1.60, 5.03
I smoke hashish/marijuana	Drinking alcohol is fun	4.46	2.59, 7.71
I take cocaine/heroin daily	I smoke not to feel excluded	6.47	1.56, 26.83
I have drunk alcohol	Drinking alcohol is normal in social situations	2.71	1.33, 5.53
I have drunk alcohol	Drinking alcohol is fun	11.09	4.13, 29.77

**Table 5:** Factor loadings (rotated component matrix of the principal component analysis).

Factor Label	1	2	3	4	5	6	7	8	9
I have smoked at least once in my life	0.730								
I currently smoke	0.875								
Cigarette consumption	−0.842								
Hashish/marijuana consumption	0.723								
Religion		0.739							
I allow smoking in my presence		0.540							
I have drunk alcohol		0.739							
Have you been drunk?		0.731							
I travelled with someone who was driving under the influence of alcohol and/or drugs		0.523							
I exercise or walk			−0.883						
I have time to do sports			−0.515						
How many times a week should you exercise?			0.804						
How many hours a day do you exercise?			0.843						
Have you ever tried cocaine?				0.752					
Have you ever tried heroin?				0.832					
Have you ever tried LSD?				0.881					
Have you ever taken drugs or pills to get high?				0.570					
Sex					0.806				
Weight					−0.784				
Height					−0.861				
It is better to enjoy sex without any contraceptive method					0.419				
If I am driving I wear a seatbelt/helmet						0.486			
Healthy nutrition improves academic performance						0.759			
Physical activity can improve health/mood						0.806			
Drinking water is important for health						0.820			
I have driven under the influence of alcohol/drugs							0.516		
I have exceeded the speed limit							0.631		
I have driven using a mobile							0.763		
Drinking alc. is normal in social situations								0.624	
Drinking alcohol is fun								0.679	
DAP is like any other birth control method								0.522	
I have dieted on the last 12 months									0.548
Thinner = Healthier									0.617
Figure is the most important									0.504

## Data Availability

Not applicable.

## Supplementary Materials

The following supporting information can be downloaded at: https://www.mdpi.com/article/10.3390/ijerph19095765/s1, Lifestyle Practices and Health Beliefs Questionnaire.
