# Supplementary material for: Analysis of the Lifestyle of Spanish Undergraduate Nursing Students and Comparison with Students of Other Degrees
Source: Int J Environ Res Public Health. 2022 May 9;19(9):5765. doi: 10.3390/ijerph19095765 (PMC9103797; doi:10.3390/ijerph19095765)
Supplement: Supplementary file 1 [file ijerph-19-05765-s001.zip › ijerph-1684024-supplementary.pdf]

LIFESTYLE PRACTICES AND HEALTH BELIEFS QUESTIONNAIRE

The following questionnaire aims to assess practices and beliefs related to healthy lifestyle. Put an "x" in the box that best describes your behavior. Answer truthfully. There are no right or wrong answers.

**Sexo: Male____ Female____**
**Your age is between: 18-20____21-25____26-30____>30**
**Degree:**
**Course: 1º___2º___3º___4º___**
**Your weight is between: 40-50___51-60___61-70___71-80___81-90___ More than 90___**
**Your height is between: 1.50-1.60___1.61-1.70___1.71-1.80___1.81-1.90___ More than 1.90___**

**A.- PHYSICAL EXERCISE: PRACTICES**

**1** - Do you exercise, walk or play sports?: **YES**___ **NO**___
**2** Do you have time to do sport: **YES** ___ **NO**___
**3** - Approximately how many days a week do you do sport?:
1 a 2 days ☐ 3 days ☐ 4 ☐ 5 or more ☐
**4** - Approximately how many hours a day do you do it?:
30 minutes ☐ 30 minutes to 1 hour ☐ 1 to 2 hours ☐ More than 2 hours ☐
**5** - Does it keep your body weight stable?: **YES** ___ **NO**___
**6** - I go to university: **walking** ___ **by car**___ by **Public transport** ___

**B.- EATING HABITS: PRACTICES**

**7** - I drink between 4 and 8 glasses of water a day.: **YES** ___ **NO**___
**8** - I eat sweets, ice cream, cakes more than 2 times a week: **YES** ___ **NO**___
**9** - I eat less than 2 rations of fruit per day.: **YES** ___ **NO**___
**10** - I eat less than 1 ration of vegetables per day: **YES** ___ **NO**___
**11**- I eat 2 or more rations of meat products per day: **YES** ___ **NO**___
**12** - I consume less than 3 servings of milk/derivatives per day: **YES** ___ **NO**___
**13** - I eat 2 or more rations of pastries per day: **YES** ___ **NO**___
**14** - I eat salty snacks (crisps, chips, snacks...) 2 or more rations per week: **YES** ___ **NO**___
**15** - In the last 12 months I have been on a weight loss diet.: **YES** ___ **NO**___
**16** - I go to university without breakfast: **YES** ___ **NO**___

**C.- USE OF TOBACCO, ALCOHOL AND OTHER DRUGS: PRACTICES**

**17** - I have ever smoked: **YES___ NO___**
**18** - I currently smoke: **YES ___ NO___**
**19** - Number of cigarettes per day:______
**20** - I allow smoking in my presence: **YES ___ NO___**
**21** - Have I ever had any of the following drinks:

	**YES**	NO
BEER		
WINE		
SPIRITS OR MIXERS		

22 - How often do you currently drink alcoholic beverages??

	All days	All weeks	All month	Less than once a month	Never
BEER					
WINE					
SPIRITS OR MIXERS					

**23** - At what age do you remember having your first drink of any alcoholic beverage?:___
**24**- Have you ever drank so much that you got drunk?:
**yes, 1 time:___** **Yes, 4-10 times:___**
**Yes, 2-3 times:___** **Yes, more than 10 times:___**
**25** - Have you ever taken these drugs??

	3 times or more	1 or 2 times	Never
Hashish, joints-marijuana			
Solvent glues			
Cocaine			
Heroine, morphine, opium			
LSD			
Medicines or pills to get high			

**D.- SEXUAL RELATIONS: PRACTICES**

**26** - At what age did you have your first sexual intercourse?:_____
**27** - I have a steady partner: **YES ___ NO___**
**28** - I use contraception in sexual intercourse with my regular partner: **YES ___ NO:___**
**29** - I use contraception in sexual intercourse with NON-stable partners: **YES ___ NO___**
**30** - I know about sexually transmitted diseases: **YES ___ NO___**
**31** - I use the morning-after pill if I do not use another method of contraception: **YES ___ NO___**
**32** -Considero haber tenido alguna vez relaciones de riesgo: **YES ___ NO___**

E.- ROAD SAFETY: PRACTICES

**33** - If I get on a motorbike, I wear a helmet: **YES** ___ **NO**___
**34** - If I get into a car as a driver or co-driver, I wear my seatbelt: **YES** ___ **NO**___
**35** - I have ever driven under the influence of alcohol/drugs: **YES** ___ **NO**___
**36** - I have ever ridden with someone in a car under the influence of alcohol/drugs: **YES** ___ **NO**___
**37** - I have ever driven with excessive speed: **YES** ___ **NO**___
**38** - I have ever driven while using my mobile phone at the same time: **YES** ___ **NO**___

A.- PHYSICAL EXERCISE: BELIEFS

**39** - Physical activity helps to improve health: **YES** ___ **NO**___
**40** - Exercising can prevent some diseases: **YES** ___ **NO**___
**41** - Exercise improves mood: **YES** ___ **NO**___
**42** - Physical exercise is useless: **YES** ___ **NO**___
**43** - Physical exercise is boring: **YES** ___ **NO**___
**44** - Since I have been exercising I have more vitality: **YES** ___ **NO**___

B.- EATING HABITS: BELIEFS

**45** - How much slimmer the more healthy you are: **Agreed** ___ **Disagree** ___
**46** - The most important thing is the body shape: **Agreed** ___ **Disagree** ___
**47** - Water is important for health: **Agreed** ___ **Disagree** ___
**48** - A good diet is important for studying performance: **Agreed** ___ **Disagree** ___
**49** - Fast food makes better use of time: **Agreed** ___ **Disagree** ___

C.- USE OF TOBACCO, ALCOHOL AND OTHER DRUGS: BELIEFS

**50** - I would like to stop smoking: **YES** ___ **NO**___
**51** - I would ask for help from health services to stop smoking: **YES** ___ **NO**___
**52** - Drinking alcohol is normal in social relationships: **YES** ___ **NO**___
**53** - Drinking alcohol is fun: **YES** ___ **NO**___
**54** - I consider illegal drugs to be easily accessible: **YES** ___ **NO**___
**55** - I consider that I have enough information about illegal drugs: **YES** ___ **NO**___

D.- SEXUAL RELATIONS: BELIEFS

**56** - Condoms are the best method of contraception: **YES** ___ **NO**___
**57** - Condoms are very uncomfortable: **YES** ___ **NO**___
**58** - Condoms prevent AIDS/STDs/unwanted pregnancies: **YES** ___ **NO**___
**59** - The morning-after pill is just like any other normal method of contraception: **YES** ___

**NO___**
**60** - It is better to enjoy sex without any method of contraception: **YES ___ NO___**
**61** - I have good information about sex in terms of STD/STD/unwanted pregnancy prevention **YES ___ NO___**

**E.- ROAD SAFETY: BELIEFS**

**62** - Traffic accidents can be avoided: **YES ___ NO___**
**63** - I see traffic accidents as a health and life problem: **YES ___ NO___**
**64** - Drugs do not affect driving: **YES ___ NO___**
